# Pathophysiology of Subjective Tinnitus: Triggers and Maintenance

**DOI:** 10.3389/fnins.2018.00866

**Published:** 2018-11-27

**Authors:** Haúla Faruk Haider, Tijana Bojić, Sara F. Ribeiro, João Paço, Deborah A. Hall, Agnieszka J. Szczepek

**Affiliations:** ^1^ENT Department, Hospital Cuf Infante Santo – NOVA Medical School, Lisbon, Portugal; ^2^Laboratory of Radiobiology and Molecular Genetics, Vinča Institute of Nuclear Sciences, University of Belgrade, Belgrade, Serbia; ^3^NIHR Nottingham Biomedical Research Centre, Nottingham, United Kingdom; ^4^Hearing Sciences, Division of Clinical Neuroscience, School of Medicine, University of Nottingham, Nottingham, United Kingdom; ^5^Queen’s Medical Centre, Nottingham University Hospitals NHS Trust, Nottingham, United Kingdom; ^6^University of Nottingham Malaysia, Semeniyh, Malaysia; ^7^Department of Otorhinolaryngology, Head and Neck Surgery, Charité – Universitätsmedizin Berlin, Corporate Member of Freie Universität Berlin, Humboldt-Universität zu Berlin, and Berlin Institute of Health, Berlin, Germany

**Keywords:** idiopathic, auditory system, pathophysiology, central tinnitus, peripheral tinnitus, causes, maintenance

## Abstract

Tinnitus is the conscious perception of a sound without a corresponding external acoustic stimulus, usually described as a *phantom* perception. One of the major challenges for tinnitus research is to understand the pathophysiological mechanisms triggering and maintaining the symptoms, especially for subjective chronic tinnitus. Our objective was to synthesize the published literature in order to provide a comprehensive update on theoretical and experimental advances and to identify further research and clinical directions. We performed literature searches in three electronic databases, complemented by scanning reference lists from relevant reviews in our included records, citation searching of the included articles using Web of Science, and manual searching of the last 6 months of principal otology journals. One-hundred and thirty-two records were included in the review and the information related to peripheral and central mechanisms of tinnitus pathophysiology was collected in order to update on theories and models. A narrative synthesis examined the main themes arising from this information. Tinnitus pathophysiology is complex and multifactorial, involving the auditory and non-auditory systems. Recent theories assume the necessary involvement of extra-auditory brain regions for tinnitus to reach consciousness. Tinnitus engages multiple active dynamic and overlapping networks. We conclude that advancing knowledge concerning the origin and maintenance of specific tinnitus subtypes origin and maintenance mechanisms is of paramount importance for identifying adequate treatment.

## Introduction

Tinnitus is a prevalent symptom associated with various conditions and diseases; both otological and non-otological (Baguley et al., 2013). It affects over 70 million people in Europe and more than 50 million people in the United States (Heller, 2003; Henry et al., 2005; Baguley et al., 2013). The heterogeneity of tinnitus causes a substantial problem in its classification, which has hampered both basic and clinical research. A large majority of people with tinnitus have experienced the symptoms for at least 3 to 6 months (i.e., chronic), and their condition has an unknown etiology (i.e., it is subjective). This review considers subjective chronic tinnitus. A major challenge for the field is to identify the underlying causes of subjective chronic tinnitus for developing specific treatments that address the distinct manifestations of tinnitus (Norena, 2015). Although much research is underway, the precise pathophysiology of tinnitus remains unclear.

Tinnitus can be classified according to various criteria including causes, comorbidities, symptoms characteristics, and psychological burden. The most common form of tinnitus is described as the conscious perception of a phantom sound or noise perceived in the ear(s) or head in absence of a known external or internal stimulus (Schlee et al., 2014) and this is often associated with a hearing loss. Tinnitus has been further classified according to its initial triggers as a *primary tinnitus*, which is either associated with sensorineural hearing loss (SNHL) or is idiopathic (or unknown cause), and a *secondary tinnitus*, which is related to other causes such as an organic origin (Tunkel et al., 2014). Somatic or somatosensory tinnitus is a subtype of subjective tinnitus, in which tinnitus perception is caused by an alteration in somatosensory afference from the cervical spine or temporomandibular area (Michiels et al., 2018). Another causal classification strategy is based on the origin of tinnitus in relation to the site of impairment in the auditory pathway, and splits tinnitus into peripheral and central types (Henry et al., 2014). Tinnitus duration is also a common symptom classification since this can distinguish patients where tinnitus is maintained over the longer term after its initial onset. *Acute tinnitus* has been defined as an onset within the past 6 months, whereas *chronic tinnitus* refers to symptoms lasting 6 months or longer (Tunkel et al., 2014). However, the precise temporal boundary from acute to chronic is not standardized, since other authors report the transition from acute to chronic tinnitus anywhere between 3 and 12 months (Hall et al., 2011; Rabau et al., 2015). Another symptom classification is based on a description of the tinnitus sound such as whether it is continuous or intermittent, pulsatile or non-pulsatile. Questions about duration and symptom characteristics are often asked in case history questionnaires (e.g., Tinnitus Sample Case History Questionnaire, Schecklmann et al., 2015).

Another classification system takes account of the functional and psychological impacts caused by tinnitus, and this is particularly important for those with chronic bothersome tinnitus. A number of questionnaires have been designed to assess self-reported impacts and examples include the Tinnitus Handicap Inventory (Newman et al., 1996), Tinnitus Questionnaire (Hallam et al., 1988), Tinnitus Functional Index (TFI, Meikle et al., 2012), and Tinnitus Primary Function Questionnaire (TPFQ, Tyler et al., 2014). The correlation between total scores of THI and TQ is 0.641 (*P* < 0.0001), indicating that they assess a similar tinnitus-related construct. Of note, the German version of the TQ (Hiller and Goebel, 1992), frequently used in the German-speaking countries, is a modified version of the original TQ developed in the United Kingdom. Burden can be represented by a score on a continuous scale, by narrative description on a categorical scale, or by a dichotomous distinction such as between “*compensated*” or “*decompensated”* tinnitus as measured by the German version of the TQ.

Whether or not any of these classification strategies are informative with respect to the pathophysiology of tinnitus remains controversial. Concerning its origin, there is a minimum consensus that tinnitus is related to aberrant neural activity at certain levels of the auditory system (Jastreboff, 1990). “Peripheral tinnitus” refers to the auditory perception that results from aberrant neural activity at the cochlear level and transmitted through the auditory pathways (Jastreboff, 1990; Guitton et al., 2003; Puel and Guitton, 2007). “Central tinnitus” refers to the auditory perception that is generated in auditory brain centers by the aberrant neural activity and is sustained by that aberrant neural activity (Eggermont, 2005, 2007; Kaltenbach, 2006, 2007; Mulders and Robertson, 2009). The auditory centers perform an important role because they are involved in the generation of the tinnitus-related activity (Liberman and Dodds, 1984a,b; Heinz and Young, 2004; Norena, 2015). Despite this distinction, “peripheral tinnitus” and “central tinnitus” are not completely independent forms (Norena, 2011). This article uses systematic review methodology to identify the latest knowledge regarding the different pathophysiological mechanisms that trigger and maintain tinnitus symptoms.

## Identifying and Selecting Appropriate Literature Sources

Eligible information sources were review articles and original research articles reporting basic science, exploratory and investigational studies. We included animal and human studies investigating tinnitus pathophysiology, but we did not include studies where the primary focus was an associated condition (such as Ménière’s disease, otosclerosis, vestibular schwannoma, chronic otitis media, tumor, autoimmune diseases, neurodegenerative or demyelinating disease, or cases of ototoxicity) with tinnitus as an incidental observation. Other exclusion criteria were articles not written in English language, and records relating solely to objective or somatosensory tinnitus.

Initial literature searches were conducted in October 2017 using three literature search platforms: PubMed, Medline and Web of Science and the search terms “pathophysiology” and “subjective chronic tinnitus.” The initial search was complemented by scanning reference lists from relevant reviews in our included records, citation searching of the included primary scientific articles using Web of Science. Additionally, in May 2018, we performed an update by manually searching key otology journals. An example search strategy for PubMed is given in Supplemental Material 1.

**FIGURE 1:**
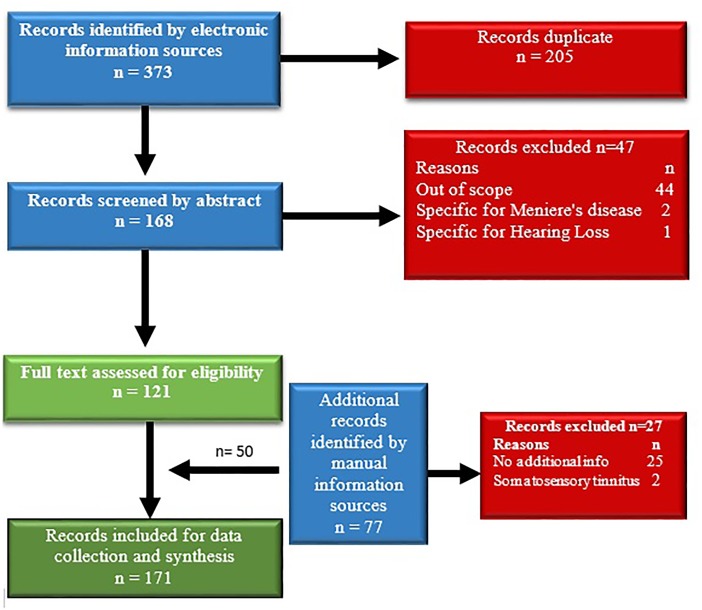
Flowchart of the literature search and selection process.

The initial search retrieved 373 records. After duplicates had been removed, 168 records remained for abstract screening. From those, 47 records were excluded as not related to the topic of the review or not meeting the inclusion criteria. The remaining 121 full texts were screened again for eligibility (Figure 1). Fifty additional records were identified by manual search resulting in a total of 171 publications. The records were then split into equal parts and the reading and data extraction was assigned to two persons working in parallel. After this step, the data were assessed by the leading authors. In case of disagreement between the extracted or interpreted data, arbitration by a third member of the team was obtained. The information extraction and synthesis focused on tinnitus pathophysiology.

## Population Characteristics Indicating Pathophysiology

A study in Italy performed by Martines et al. (2010a,b,c) estimated that in 30% of cases, tinnitus had an undetermined etiology. It is well established that tinnitus often accompanies noise-induced hearing loss and presbycusis. According to Davis and Rafaie (2000), approximately 90% of people with tinnitus in the United Kingdom have some form of hearing loss. Large-scale population studies have identified other risk factors such as vascular disease, hypertension, diabetes, autoimmune disorders, head injury, and degenerative neural disorders (Rojas et al., 2003; Sindhusake et al., 2004).

## Comparing Animal and Human Neurophysiological Studies

Some of the major advantages of the animal model as a way to investigate the pathophysiology of tinnitus are the ability to (i) control the etiology via controlled experimental manipulation of the noise environment or ototoxic drug exposure, (ii) to randomly assign animals to experimental or control groups, increasing the power of statistical testing, and (iii) to apply a wide range of experimental tools (from molecular to behavioral). Nevertheless, some disadvantages of using animals for tinnitus research exist, the main one being the lack of a standardized animal model of tinnitus. These fundamental challenges give rise to concerns about the reliability and interpretation of results (Lobarinas et al., 2013; Brozoski and Bauer, 2016). Noise exposure in the animal model is often traumatic and acute, unlike the more common human experience of moderate and prolonged noise exposure, while exposure to highly concentrated ototoxic agents such as salicylate are rare in humans. An unresolved issue is the distinction between acute and chronic tinnitus in animal models, mainly due to different experimental paradigms and different species used. An agreed classification of what constitutes acute versus chronic tinnitus in the animal model is of special importance for future studies regarding the progression from acute to chronic forms, especially since this could provide the basis for seeking objective markers of its natural history. The majority of research done with help of animal models points to noise-induced hearing loss and tinnitus as an adequate model for the development of chronic tinnitus (Bauer and Brozoski, 2001; Turner and Larsen, 2016). The report of Pace et al. (2016) focuses on a novel experimental paradigm and makes distinction between the salicylate-induced tinnitus (tinnitus duration 5 days) and noise-induced tinnitus (tinnitus duration 7 weeks). An attempt to define such criteria has already been made using clinical studies (Leaver et al., 2016a). Based on the obtained findings, species-specific criteria could be expected to emerge in animal models of tinnitus.

The pioneering and widely applied salicylate model (Jastreboff et al., 1988) induces tinnitus both by direct central effects on the auditory system and by induction of peripheral hearing loss (Eggermont, 2015). For a detailed review on animal models of tinnitus, the reader could refer to Brozoski and Bauer (2016). Questions about altered neural spontaneous firing rates in the auditory pathway, abnormal neural synchrony and changes in tonotopic representation have been obtained from animal studies at the level of individual neurons and neuronal assemblies, and in human studies at a much more macroscopic population level (Adjamian et al., 2009; Eggermont, 2015). The main problem here is the translation of research from subcellular neuronal events found in animal models to the brain activity patterns observed in people with tinnitus. The differences in measurement technique bring important caveats for drawing analogies between animal and human findings. For example, the assumption that the interpretation of coupling between local neural activity and the responses monitored using blood oxygenation level-dependent functional magnetic resonance imaging (BOLD-fMRI) are still unclear (Adjamian et al., 2009).

One of the overall impressions about the neurophysiological results obtained from animal models of tinnitus is that they typically consider tinnitus as the consequence of an acute peripheral lesion associated with severe hearing loss. In contrast, human neuroimaging studies tend to emphasize the role of auditory thalamus and auditory cortex in the chronification and maintenance of tinnitus (Eggermont, 2015; Brozoski and Bauer, 2016).

## Sites of Tinnitus Generation

A fundamental question in tinnitus pathophysiology concerns the neural component that generates tinnitus (Henry et al., 2005). Zenner (1998) initially postulated that tinnitus could originate in any relevant anatomical structure; from the ear throughout the central auditory pathways. Initial speculations favored a cochlear origin since tinnitus can be perceived in the ears and also due to the fact that there is a strong association between the frequency of psychoacoustic identified tinnitus and the audiometric profile of hearing thresholds (Sereda et al., 2011). These opinions were contradicted by the fact surgical section of the auditory nerve does not eliminate tinnitus in every case, which favors the hypothesis about the central rather than peripheral origin of tinnitus (House and Brackmann, 1981).

Nowadays, it is well established that many forms of tinnitus reflect a complex interaction between peripheral and central mechanisms within the auditory pathway (Norena and Farley, 2013). Usually two or more triggers (e.g., noise exposure, hearing loss, emotional distress, and somatosensory factors) are necessary to elicit a noticeable tinnitus (Shore et al., 2007). Tinnitus can be seen as a pathology of neural plasticity with a molecular and a systemic component. The molecular component has a cochlear component related to the initiation phase of tinnitus; while the systemic component has a central aspect associated to the long-term maintenance of tinnitus (Satar et al., 2003; Guitton, 2012; Norena and Farley, 2013; Norena, 2015; Sedley et al., 2015). It has been suggested that peripheral tinnitus may originate from the dysfunction of cochlear outer hair cells (OHCs) and the consequent changes in endocochlear potential, leading to increased spontaneous cochlear activity. This suggestion provides a possible explanation of different causes behind cochlear tinnitus, including tinnitus induced by an acute noise exposure (Norena, 2015). Meanwhile, central tinnitus is mediated by the neuronal activity in the auditory centers. A good illustration is the chronic tinnitus induced by a noise trauma in the absence of changes in cochlear activity following the trauma (Liberman and Dodds, 1984a,b; Heinz and Young, 2004; Norena, 2015). Although central mechanisms are important for explaining the generation of tinnitus-related activity, much of these mechanisms appear to be triggered by a reduction of cochlear activity. However, damage to cochlear tissues is not necessary to produce central changes related to tinnitus, since a conductive hearing loss can also induce tinnitus (Ayache et al., 2003; Midani et al., 2006; Schaette et al., 2012).

Based on the above assumptions, Norena (2015) proposed three distinct subtypes of tinnitus: cochlear tinnitus, peripheral-dependent central tinnitus, and peripheral-independent central tinnitus. Cochlear tinnitus refers to a tinnitus generated by aberrant activity in the inner ear, which is propagated through the cochlear nerve and the central auditory pathway. This activity may lead to an auditory perception, depending on the firing neuronal rates and top-down modulation (Norena and Farley, 2013; McKenna et al., 2014; Norena, 2015). Peripheral-dependent central tinnitus refers to a tinnitus associated with cochlear spontaneous activity, while peripheral independent central tinnitus refers to a tinnitus that is independent from cochlear spontaneous activity (Norena, 2011, 2015).

## Cellular Mechanisms

Cochlear damage may include loss of OHC electromotility, loss of synapses between Inner Hair Cells (IHCs) and spiral ganglion neurons (synaptopathy), damage to the stereociliar bundle, death of OHCs or IHCs, or rupture of the basilar membrane. All of these processes can be seen in rodents by means of histology, but are not easily measurable in humans due to difficulty in access to tissue. These mechanisms lead to a decrease in neuronal output from the cochlea to the brain and they could account for the potential generation of compensation mechanisms in the brain (Chen and Fechter, 2003).

## Position of the Tectorial Membrane

Change in the position of the tectorial membrane may be a pathophysiological trigger for acute tinnitus following an intense noise exposure. It is well established that after noise trauma, the rootlets of stereocilia are altered leading to stiffness and contributing to acute increase in cochlear spontaneous activity (Liberman and Dodds, 1984a,b, 1987). The prolonged depolarization of IHCs can occur through any condition that changes the relative position of the tectorial membrane. This may originate after an increased pressure in the scala media, tectorial membrane detachment, degeneration of OHCs or stereocilia (LePage, 1989). In some cases, there might exist areas of damaged OHCs but intact IHCs, and so the tectorial membrane can touch the IHCs stereocilia, consequently causing their depolarization (Baguley, 2002).

## Outer Hair Cells (OHCs)

Another pathophysiological trigger for acute tinnitus concerns damage to the stereocilia of OHCs, again often following an intense noise exposure. High noise levels damage first the OHCs and then the IHCs (Nicolas-Puel et al., 2006). The initiation of pathological process starts at the stereocilia of OHCs, with two fundamental processes damaged by the noise: intracellular calcium levels and biochemical changes of their structural proteins. Eggermont suggested that increased intracellular calcium could be the pathological substrate of peripheral tinnitus, by increasing the neurotransmitter release of the cells and subsequent activity of afferent fibers (Eggermont, 2000).

## Inner Hair Cells (IHCs) and the Cochlear NMDA Receptors

The *N*-methyl-D-aspartate (NMDA) receptor has been found to play an essential role in noise-induced tinnitus. In a behavioral animal model, pharmacological interventions that antagonize the NMDA receptors prevent tinnitus (Guitton et al., 2003). These NMDA receptors appear to predominate on the modiolar side of IHCs (Pujol et al., 1992), with a higher percentage of lateral olivocochlear efferent fibers that seem to terminate on low-SR high threshold fibers (Liberman, 1980). It seems that an increase in glutamate levels derived from IHCs, activates the NMDA receptors that release excessive Ca^2+^ in the dendrites of the spiral ganglion neurons. This causes an over-excitation of NMDA-receptors and consequently a calcium influx during the damage. This process may contribute to hearing loss, neural presbycusis and tinnitus via the aberrant excitation of the auditory nerve (Sanchez et al., 2015). Underlying the over-excitation, there is an increase in adenosine triphosphate (ATP) which consequently increases the reactive oxygen species in the synapses between IHCs and spiral ganglion neurons (Sahley et al., 2013). An increase in levels of Ca^2+^ in the NMDA receptors can trigger a successive metabolic events such as production of reactive oxygen or hydrogen species or even death of spiral ganglion neurons (Parsons and Raymond, 2014). It is likely that the blockade of NMDA-receptor activation prevents the loss of IHC ribbons after noise damage (Bing et al., 2015). Therefore, concerning the lower auditory pathway, the NMDA receptor plays a role in numerous functions such as neuronal plasticity, synapse modifications, temporal processing, and onset of disease (Sanchez et al., 2015).

## Increase of the Endocochlear Potential

The endocochlear potential is a prerequisite for auditory signal transduction. It is maintained by keeping high concentrations of K+ in the endolymph and is strongly associated with cochlear spontaneous activity (Sewell, 1984; Mittal et al., 2017). An increase in the endocochlear potential can depolarize IHCs, which triggers a sequence of events that includes opening the voltage-gated Ca_2+_ channels, an intracellular influx of Ca_2+_ and fusion of the synaptic ribbon to plasmatic membrane. This culminates in glutamate release and depolarization of cochlear fibers (Hudspeth, 1985; Moser et al., 2006). OHCs can regulate the endocochlear potential, through their mechano-electrical transduction channels. In other words, the opening of these channels depends on stereociliar bundle deflection. This process seems to be induced by acute noise trauma that reduces the opening probability of these channels, consequently increasing the endocochlear potential (Patuzzi, 2002).

Biochemical changes seem to be most relevant to the acute phase of tinnitus. The heat-shock protein group (stress proteins), interacts with structural proteins of hair cells, giving them support and protecting them from further damage. Any disturbance that causes a deficient heat-shock protein system response can lead to incurring tinnitus to the person exposed to loud noise (Dechesne et al., 1992).

## Cochlear Synaptopathy

Although the majority of people with tinnitus have a clinically measurable hearing loss, a good number do not. According to different series more than 60% of people with normal hearing (based on tonal audiometry) have tinnitus (Heller and Bergman, 1953; Tucker et al., 2005). Animal data suggest that the permanent loss of synapses between the IHCs and the cochlear nerve fibers occurs because external factors such as noise exposure or aging (Kujawa and Liberman, 2009; Sergeyenko et al., 2013; Kujawa and Liberman, 2015). This condition is popularly called “hidden hearing loss” (HHL) (Schaette and McAlpine, 2011), since it is not possible to diagnose through conventional tonal audiometry using quiet sounds. In the ear, noise overexposure causes a rapid excessive release of the neurotransmitter glutamate from electron-dense ribbon synapses in the IHC. This excitotoxic insult induces the swelling of the dendrites, which causes an important level of hearing loss at a particular frequency due to a partial disconnection among the IHCs and the afferent neurons (Pujol et al., 1993). The ear possesses a remarkable healing capacity that allows these neuronal terminals to regrow toward the sensory cells and reestablish functional connections restoring hearing (Pujol and Puel, 1999), as people experience after noise exposure (e.g., concerts) and have their hearing thresholds recovering and their tinnitus disappearing after some time. However, in some cases, even if the terminals have grown back, the reconnection can be incomplete and synaptic coupling remains incomplete due to either a decrease in the number of ribbons (Ruttiger et al., 2013) or a decrease in the number of paired pre-and post-synaptic entities (Kujawa and Liberman, 2009). The damage seems to selectively affect low spontaneous rate of the cochlear neurons responsible for high thresholds and coding moderate-to-high sound intensities (Furman et al., 2013). Recently, Wan and Corfas (2017) reported another mechanism underlying HHL. The authors found that transient Schwann cells loss results in permanent disruption of the cochlear heminodal and consequently in permanent auditory deficits characteristic of HHL. Interestingly, this auditory deficits is not related to the synaptic loss, but with the affection of the first heminodes at the auditory nerve peripheral terminal. This study provides new insights on the mechanisms, causes and long term consequences underlying HHL.

The extent to which cochlear synaptopathy contributes to tinnitus in animals and in humans is still uncertain. Schaette and McAlpine (2011) first demonstrated the reduced amplitude of wave 1 in the auditory brainstem response (ABR) in the subjects with tinnitus but with normal audiogram, when compared to controls. An appealing interpretation of these findings is that they are evidence for reduced cochlear nerve output as a direct result of cochlear synaptopathy. However, there are some important caveats to data interpretation. First, the match between tinnitus and control groups was not 100% regarding the high frequency sensitivity, yet wave 1 ABR amplitude is known to be predominantly raised by responses to high-frequency tones (Don and Eggermont, 1978). Second, this finding has not withstood replication (Gilles et al., 2016; Guest et al., 2017). Methodological differences might underlie the lack of replication, but another plausible explanation is that tinnitus in young audiometrically normal adults is not related to cochlear synaptopathy but may reflect other effects of the exposure to noise (Guest et al., 2017). Clear directions for further research are to improve the sensitivity of non-invasive electrophysiological measures of cochlear synaptopathy in humans, and to examine the broader neurophysiological impacts of noise exposure.

## Mechanisms Involved in Maintenance of Tinnitus

The link between hearing loss and tinnitus is well substantiated. For example, patients with conductive hearing loss (e.g., otosclerosis) frequently report having tinnitus and these symptoms are usually abolished after surgery (Gersdorff et al., 2000; Ayache et al., 2003; Sobrinho et al., 2004). Ear plugging is a way to induce a temporary hearing loss in otherwise normally hearing people. Participants who wear a silicone earplug for 7 days develop tinnitus symptoms, which disappear after removing the earplug (Schaette et al., 2012). Implantable and non-implantable hearing devices improve tinnitus in 50% of treated patients and eliminating it in 20% of cases (Schaette, 2013), likely by partially restoring cochlear output. More specifically, published data confirm a strong association between high-pitched tinnitus and high-frequency SNHL, suggesting again that hearing loss is a main cause of tinnitus (Norena et al., 2002; Martines et al., 2010a,b,c; Sereda et al., 2011). Many theories suggest that the underlying cause of tinnitus may be associated with damage to the sensory cochlear epithelium (Henry et al., 2005), and if acute then this can be assessed in the patient by asking about the temporal association between noise exposure events, abrupt changes in hearing and tinnitus onset or exacerbation. In a review, Zhao et al. (2016) found that specific insults to the peripheral auditory system (e.g., cochlear ablation, selective IHC or OHC loss, and mixed or incomplete IHC and OHC injuries) can all reduce cochlear output. The edge theory of tinnitus proposes having cochlear disturbance inducing tinnitus and caused by the shift of OHCs in the organ of Corti from the apical side toward the lesion in a high-frequency basal side (Nuttal et al., 2004). In almost all types of peripheral insults, OHCs are more damaged than IHCs. Combined with the edge theory, this provides the foundation of the Discordant Theory, which predicts that tinnitus is associated with a disinhibition of neurons in the dorsal cochlear nucleus (DCN), due for example to DCN receiving excitation from IHC and not from damaged OHC and consequently leading to increasing spontaneous activity in the central auditory system (Levine, 1999; Jastreboff and Hazell, 2004).

Reduced cochlear output through hearing loss likely triggers a cascade of neuromodulatory events ultimately causing hyperactivity in central auditory circuits (central gain). This process has been proposed to contribute to tinnitus. It seems to be associated with neuronal hyperactivity and could likely be a common consequence of various kinds of cochlear damage (Parra and Pearlmutter, 2007). It could also explain individual cases of tinnitus without hearing loss, since there can be up to 30% damage to the OHCs before hearing loss is detectable using pure tone audiometry (Chen and Fechter, 2003).

**FIGURE 2:**
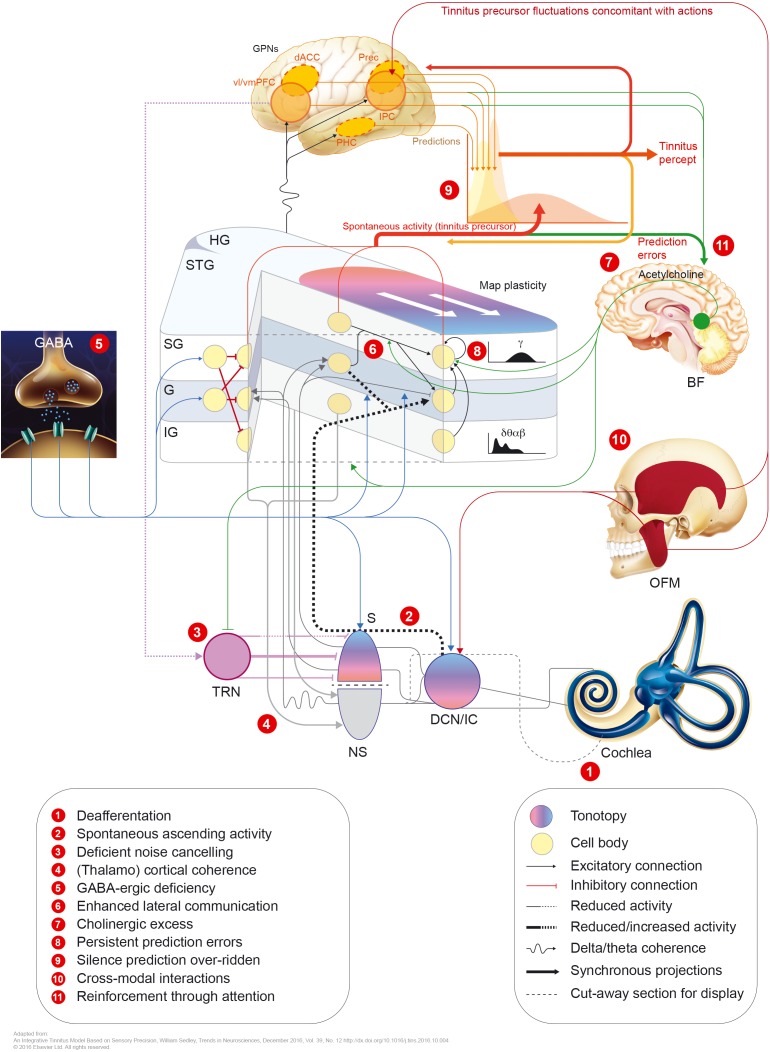
Potential mechanisms involved in tinnitus pathophysiology. GPNs, global perceptual networks; vl/vmPFC, ventrolateral/ventromedial prefrontal cortex; dACC, dorsal anterior cingulate cortex; Prec., precuneus; IPC, inferior parietal cortex; PHC, parahippocampal cortex; HG, Heschl’s gyrus; STG, superior temporal gyrus; SG/G/IG, supragranular/granular/infragranular neuronal layers; BF, basal forebrain; OFM, orofacial movements; S, specific (lemniscal) auditory thalamus; TRN, thalamic reticular nucleus; NS, non-specific auditory thalamus; DCN, dorsal cochlear nucleus; IC, inferior colliculus.

Hearing loss decreases the input to the central auditory system. This may in turn modify the gain of central neurons, resulting in increased spontaneous activity. The functional aberrations resulting from either model (tonotopic over-representation, enhanced synchronicity, or elevated spontaneous firing rates) may underlie the induction of tinnitus (Adjamian et al., 2014; see Figure 2).

**FIGURE 3:**
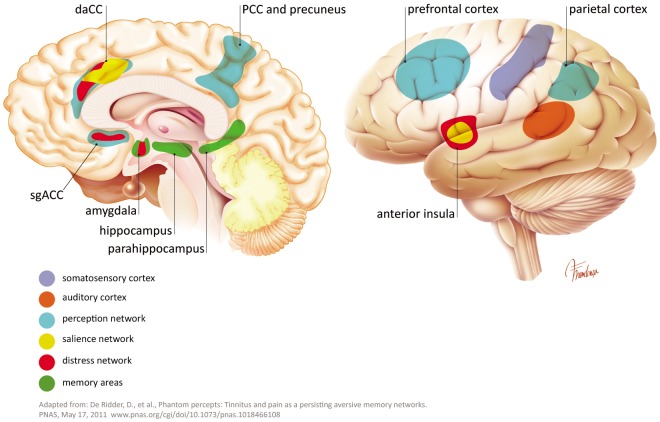
Some extra auditory regions involved in tinnitus pathophysiology.

The sensation of pain and phantom limb perception is often used as an analogy to the pathophysiology of tinnitus. Damage in the cochlea (e.g., hair cell loss or synaptic damages) leads to a frequency-specific decrease in output from the cochlear nerve. An upregulation of activity in the central auditory pathway is a compensatory effort to counteract the lack of signals in the particular frequency area. This effort increases the gain, falsely leading to the perception of a non-existing sound and possibly accompanying hyperacusis (Auerbach et al., 2014). In addition to the auditory pathway, tinnitus shares non-auditory networks, similar to these know in chronic pain (perception, salience, distress, and memory). Such networks, may maintain, in absence of the initial “tinnitus-initiator” (De Ridder et al., 2011a, 2014; Rauschecker et al., 2015). De Ridder and others consider phantom pain and phantom sound to share basic underlying mechanisms. The model assumes sensory deafferentiation resulting in cortical activity within the primary and secondary auditory cortices. This activity becomes a conscious percept upon connection to a larger brain networks located in the frontal and parietal areas of cortex, such as “self-awareness” and “salience network.” The latter network intersects with the central autonomic control system and affects the limbic-auditory and somatosensory interaction indispensable for consciously maintaining the phantom perception (see Figures 2, 3). This perception may associate with distress, simultaneously co-activating non-specific distress networks located in the anterior cingulate cortex, anterior insula and amygdala. At the same time, it is proposed that memory mechanisms may reinforce and maintain the awareness of the phantom percept (De Ridder et al., 2011a).

## Central Mechanisms

The compensation mechanism occurring in the central nervous system during tinnitus is called “homeostatic plasticity.” This is a phenomenon whereby auditory neurons in the brain adapt their synaptic connections in attempt to maintain a neuronal network similar to the one before the peripheral damage occurred. Neuronal correlates of tinnitus have been proposed as neuronal hyperactivity in the posteroventral cochlear nucleus (PVCN), the inferior colliculus (IC), DCN, and the paraflocculus lobe of the cerebellum (PFL) (Cacace et al., 2014). Specifically, it has been suggested the presence of elevated responses to sound in subcortical areas, in particular in the IC, as a common effect among individuals with tinnitus and normal thresholds (Melcher et al., 2009). A large body of data supports the view that DCN is the induction site of tinnitus, which then spreads to higher areas (Brozoski et al., 2012; Dehmel et al., 2012; Wu et al., 2015). Animal studies show an increased activity in fusiform neurons of the dorsal cochlear nucleus during noise-induced tinnitus (Brozoski et al., 2002). Being the site of convergence of different somatosensory pathways (trigeminal nucleus and dorsal somatosensory pathway), cholinergic and serotonergic systems, it has been proposed that the DCN is an important site of maladaptive auditory-somatosensory plasticity (Wu et al., 2015). Supporting the importance of the DCN in tinnitus generation is the identification of a role of the Kv7.2/3 channel, which shows decreased activity in the DCN after noise-induced tinnitus. However, a specific drug compound that modulates Kv channels (Kv3.1)^1^ has been found not to alleviate subjective tinnitus in humans^2^.

Another hypothesis views tinnitus as a product of neuronal hyperactivity in particular regions of the central auditory system such as cochlear nucleus, IC and thalamus – see Dong et al. (2010), Middleton et al. (2011), Vogler et al. (2011), Manzoor et al. (2013) and Kalappa et al. (2014). There is no consensus about cannabinoids, which activate the CB1 receptors and which may have an effect on exacerbation or worsening tinnitus. However, the presence of CB1 receptors in the DCN was suggested to increase rather than to inhibit tinnitus (Smith and Zheng, 2016).

There are two main, partially compatible theories on the role of medial olivocochlear bundle in tinnitus onset. The first theory emphasizes the role of decreased neural efferent input to the cochlear amplifier which, in this way, increases its spontaneous activity and induces a chain reaction of neuroplastic changes in the afferent auditory relays up to the auditory cortex. The second theory focuses on the brainstem as the place of integration of efferent neuronal drive and afferent tinnitus-related stimuli (Riga et al., 2015). Considering that some studies could not confirm the role of medial olivocochlear bundle in tinnitus, this finding is still controversial (Riga et al., 2015).

The auditory cortex also shows evidence of frequency-dependent reorganization, although in people with tinnitus but without measurable hearing loss, tonotopic map reorganization is not essential (Langers et al., 2012). Oscillatory activity (periodic fluctuations in electromagnetic field/potential as a result of synchronized firing of large neuronal ensembles) is one method for measuring neural synchrony in the human brain. The power of the oscillatory activity can be separated into different frequency bands, namely: delta (1–4 Hz), theta (5–7 Hz), alpha (8–12 Hz), beta (13–20 Hz), and gamma (>30 Hz). The premise is that these reflect different functional processes.

Comparing cortical hubs that involve multiple brain regions in people with tinnitus and in the healthy controls through electrophysiological measurement demonstrates fundamental differences between the groups (Muhlnickel et al., 1998; Schlee et al., 2009). Mapping the cortical hubs has demonstrated essential differences in the global networks, mainly hyperactivity in the gamma frequency range within the temporal cortex associated with tinnitus (Schlee et al., 2009). According to this view, the global network may influence the auditory cortex in a top-down process and regulate the degree of tinnitus-related distress. Those alterations seem to be associated with conscious tinnitus perception (Schlee et al., 2008). In particular, the activity and connectivity patterns detected in the posterior cingulate cortex and the precuneus region, associate with a distressing tinnitus (Maudoux et al., 2012). When cochlear damage causes a reduction of electric signals at a given frequency, neurons within the primary auditory cortex responsive to these frequencies start responding to adjacent frequencies, as exemplified by the broadening of the frequency tuning in this region (Engineer et al., 2011; Yang et al., 2011). Aberrant neuronal oscillations have also been observed in the alpha and gamma frequency range within the frontal cortex (Muller et al., 2013). These results are in agreement with the work of Weisz et al. (2005), who were the first group to use the electroencephalography (EEG) oscillation to study tinnitus. That first study revealed the dissimilarities of power spectra between a group of people with tinnitus and hearing loss and a matched group of control subjects. Over the years, other results provided mixed support for this finding (Weisz et al., 2007; Moazami-Goudarzi et al., 2010; De Ridder et al., 2011b; Adamchic et al., 2012, 2014; Adjamian et al., 2012), and there is not yet any clear agreement in the field. For example, recently, Pierzycki et al. (2016) found no evidence that resting state whole-scalp EEG reflects any tinnitus-related percept or symptom severity and so should not be assumed as a biomarker for tinnitus. Moreover, the correlation between perception of tinnitus and the frequency band power in EEG and magnetoencephalography (MEG) remains unclear. Using acoustic stimulation to test residual inhibition (RI) when tinnitus is reduced, both delta/theta and gamma are suppressed (positive correlations); when tinnitus is louder – residual excitation (or Rebound Effect) (RE) delta/theta is unchanged and gamma is reduced (negative correlation) (Sedley et al., 2012).

Overall, it is now rather well established that most of the nuclei in the auditory pathway can be affected during tinnitus. These compensatory mechanisms seem to be related to the loss of GABAergic inhibition and decreased activity of specific potassium channels (Kv7.2/3) (Yang et al., 2011; Li et al., 2013). However, whether the changes seen in central gain are directly related to tinnitus or instead more related to hyperacusis is still a matter of discussion (Knipper et al., 2013; Auerbach et al., 2014).

## Non-Auditory Neuronal Networks Involved in Tinnitus

Recent work in rodents (with fMRI) and humans (intracranial recordings) strongly support the involvement of emotional/cognitive relays of the brain such as temporal, parietal, sensorimotor, and limbic cortex in the pathophysiology of tinnitus (Frank et al., 2011; Vanneste and De Ridder, 2011). Neuronal emotional networks which influence peripheral and central circuits during tinnitus, involve most likely central regions implicated in a normal emotional behavior and in mood altered disorders. Such regions comprise the medial prefrontal cortex and ventromedial parts of the basal ganglia (also known as limbic frontostriatal network) (Lowry et al., 2004; Cheung and Larson, 2010). In addition, they include dorsal prefrontal regions, the medial and caudolateral orbital cortex (medial prefrontal network), insula, posterior thalamus, anterior cingulate, posterior cingulate, amygdala (Shulman, 1995; Mirz et al., 2000), parahippocampus, hippocampus (Lockwood et al., 1998; Landgrebe et al., 2009), and the subcallosal region (Muhlau et al., 2006; Leaver et al., 2011) including the nucleus accumbens (Jastreboff, 1990; Drevets et al., 2008). The precise functional role of the numerous extra-auditory structures is difficult to establish because some of them participate in the generation or in the chronification of tinnitus, some in psychological reactions to the tinnitus, some are associated with hearing loss and others with hyperacusis (Leaver et al., 2016a,b; see Figures 2, 3). It is highly plausible that there is no coherent model for the involvement of extra-auditory structures in chronic tinnitus but rather that the patterns are highly dependent on the individual tinnitus profile. A tight interaction between limbic non-auditory and auditory pathways and the presence of both anatomical and functional abnormalities has been confirmed by different neuroimaging techniques (stimulus evoked BOLD fMRI, diffusion MRI, resting-state fMRI and PET) (Leaver et al., 2016b). On the other hand, other groups have not been able to determine significant differences in the connectivity of auditory network between control and tinnitus groups (Davies et al., 2014). One of the important observations is that the involvement of the extra-auditory brain areas traces the evolution of acute tinnitus to its chronic form (Leaver et al., 2016b). Because a relationship between the psychoacoustic tinnitus characteristic, the degree of tinnitus distress and underlying neural patterns of activity is not scientifically confirmed, there is an urgent need for systematic studies to address these questions further (Leaver et al., 2016b).

The frontostratial circuits appear to have a central role in the development and maintenance of both tinnitus and chronic pain (Rauschecker et al., 2015). Two structures are essential in this process: the ventromedial prefrontal cortex and the nucleus accumbens. Both of them play a role in evaluating the relevance and emotional significance of sensory stimuli and in managing of the information flow via descending pathways. The damage in frontostratial areas could explain tinnitus pathophysiology and provide new insights for the therapeutic design or prevention of tinnitus and chronic pain (Rauschecker et al., 2015). The tinnitus percept seems to be mediated by a somatotopic map and the corresponding somatic memory. Furthermore, somatic memories depend on somatotopic maps and their active use in the specialized cortical areas (Eggermont and Kral, 2016). The genetically defined somatic memories and the somatotopic maps are shaped by experience during early development, and are independent of auditory input (Bonham et al., 2004; Pienkowski and Harrison, 2005; Eggermont and Moore, 2012). Corroborating this observation, it was noted that the individuals born without limb(s) are free of phantom limb and phantom limb pain phenomena. This observations reinforces the relationship between tinnitus and the phantom limb that occurs as references to sensory surface maps (Eggermont and Kral, 2016).

The medial geniculate body (MGB) within the thalamus has been suggested to gate the perception of sound on its way to the auditory cortex and to limbic system (Caspary and Llano, 2017). The key component in the pathology of the tinnitus network strongly implicates MGB and its ascending inputs from the brainstem, thalamic reticular nucleus and, limbic structures, as well as descending inputs from the auditory and non-auditory cortices (Shinonaga et al., 1994; Bajo et al., 1995; Lee and Winer, 2008a,b,c; Rauschecker et al., 2010; Leaver et al., 2011). In addition, a functional model of tinnitus suggests that in the affected individuals, tinnitus-related distress correlates with abnormal functions in limbic and thalamocortical circuits (Winer et al., 1999; Rauschecker et al., 2010; Leaver et al., 2011). Concerning the role of MGB, opposing hypotheses offered GABA-related explanations. The first one assumes tinnitus-related up regulation of GABAergic inhibition whereas the second one assumes tinnitus-related suppression of GABAergic inhibition. GABA mediates fast synaptic inhibition and a persistent tonic inhibition (Caspary and Llano, 2017), indicative of the increase in GABA being alleged to increase bursting, thus, increasing thalamocortical activation.

One study has evaluated the cortical benzodiazepine receptor distribution in patients with tinnitus, using venous blood samples after radiolabeling with 123I-iomazenil, radiochemical purity, single-photon emission computed tomography (SPECT) and MRI. A comparison of participants with severe chronic tinnitus and controls revealed a significant trend toward bilaterally reduced benzodiazepine receptor density in the frontal lobes (*p* < 0.001) and a reduction in the cerebellum (*p* = 0.045) (Daftary et al., 2004).

An MRI study, involving people with hearing loss affected or not by tinnitus, demonstrated increased gray matter in the temporal and limbic areas, and decreased gray matter in frontal and occipital areas when compared to a control group. In detail, analyses of all cortical areas of the tinnitus participants demonstrated an increase of gray matter in cerebellum and subcortical auditory nuclei with the most significant effect in the left primary auditory cortex when compared to controls and those with hearing loss only. On the other hand, people with hearing loss had decreased gray matter in frontal areas and increases in limbic areas, compared to controls. These findings imply a particular role for the left primary auditory cortex and other non-auditory brain structures in tinnitus development (Boyen et al., 2013). Another study, with a similar design, using diffusion tensor imaging and voxel-based morphometry (VBM), found both gray and white matter changes in the auditory cortex of people with hearing loss but without tinnitus, compared to people with tinnitus and controls. Thus, the authors concluded that hearing loss rather than tinnitus was associated with the observed changes (Husain et al., 2011). A large-scale study examining VBM and surface-based morphometry changes in brain anatomy from 128 participants with tinnitus and hearing loss, tinnitus with clinically normal hearing, and non-tinnitus controls with clinically normal hearing managed to replicate some of the morphological differences that had been reported in previous studies, but found other differences that contradicted previous results (Allan et al., 2016). The variability of morphometry results obtained by different teams and by different analysis methods is confusing. It perhaps indicates the need for greater standardization in study design, and in analysis techniques, as well as more precise subtyping of the condition.

## Theories and Models of Tinnitus Pathophysiology

A recent report supports the notion that tinnitus is not associated with increased metabolic activity in localized auditory regions (Geven et al., 2014), but rather with neural synchrony between different cortical networks (Norena and Farley, 2013; Sedley et al., 2015), including the thalamus (Eggermont, 2013; Husain and Schmidt, 2014). A steep audiometric edge between regions of normal and impaired hearing may be sufficient to disrupt the normal pattern of neural synchrony in tonotopically organized regions of the central auditory system. De Ridder et al. (2015) have observed that oscillatory activity in the gamma frequency band usually appears bilaterally in tinnitus patients and they have proposed this to be the substrate of tinnitus. However, the evidence only partially supports this model because there are a number of methodological issues that complicate the attribution of findings to the tinnitus versus the hearing loss (Adjamian et al., 2012). With respect to this edge region, some studies have found tinnitus-related changes in the magnitude of the oscillatory power in delta/theta, alpha and in gamma frequency bands (Eggermont and Tass, 2015). These authors observed tinnitus-related low-frequency delta oscillation that are hypothesized to originate from the thalamus low frequency bursting (Sedley et al., 2015). The delta activity extended beyond auditory cortex to the temporal, parietal, sensorimotor, and limbic cortices. The diffuse distribution of activity was too extensive to be consistent with the putative “edge effect” theory. Rather, delta frequency band activity has been found to interact with alpha, beta, and gamma frequency band activities in specialized brain regions such as parahippocampal and inferior parietal regions. And this has been proposed as a neurophysiological correlate of the network-based interactions between tinnitus perception and memory processes. In line with further development of the synchronicity model, Schlee et al. (2014) investigated the correlation between chronic tinnitus and cortical activity in the alpha frequency range. The authors confirmed the reduction of alpha power and auditory alpha variability in the tinnitus brain. According to their conclusions, changes in alpha power reflect the enhanced and reduced excitability of engaged neuronal networks (Schlee et al., 2014).

Overall, these results suggest a role for neural synchrony both for establishing pathological activity within the auditory cortex and for recruiting extra-auditory networks in tinnitus. However, the precise details of these mechanisms warrants further attention.

Recently, Sedley et al. (2016) proposed another framework to explain tinnitus pathophysiology from the ear to the cortex. That model assumes a so called predictive coding model, in which spontaneous activity of the auditory subcortex involves “tinnitus precursor,” which is normally ignored against the prevailing percept of “silence” (see Figure 2). This model explains the simple and unitary content of tinnitus. The sensory precision tinnitus model comprises causes of spontaneous sensory input and their graded processing in a predictive coding framework.

The broader framework is equally applicable to other conditions similar to tinnitus, such as chronic nociceptive pain. Nevertheless, some types of pain such as central post-stroke pain, cannot be explained by this framework (Klit et al., 2009).

There are a number of psychological models of tinnitus. The neurophysiological model (Jastreboff, 1990) proposes that fear is a conditioned responses that is responsible for generating a bothersome tinnitus (Jastreboff and Hazell, 1993). The neurophysiological model draws on behavioral psychology and has the following stages: (1) generation of the tinnitus-initiating signal in the peripheric auditory system; (2) detection of the neuronal activity induced by tinnitus; (3) perceptional evaluation of tinnitus. Husain proposed a neuropsychological model that includes the regions and connections involved in mediating chronic tinnitus (Husain, 2016). The brain regions identified incorporate the neuropsychological (Jastreboff, 1990; Kaltenbach, 2006; Eggermont and Roberts, 2012) and psychological (e.g., Sweetow, 1986; Hallam et al., 1988) components of tinnitus. This model differs from those already existing in that it uses MRI evidence to explain habituation to tinnitus. The model predicts a key role of the amygdala in a severe, non-habituated tinnitus. The frontal cortex becomes more engaged in subjects with mild, habituated tinnitus, and this may facilitate bypassing the emotional processing from the amygdala and the use of alternate limbic pathways involving the insula and parahippocampus gyrus (Husain, 2016).

Tinnitus models that are influenced by the cognitive psychology movement include the cognitive behavioral model (McKenna et al., 2014) and fear-avoidance model (Cima, 2018). Both of these seek to explain the causes and chronicity of tinnitus-related distress from a cognitive perspective, and both offer an integrative approach that could shed insights on higher-order pathological processes of tinnitus-related distress.

## Conclusion

Significant advances in understanding the molecular, cellular, and system-level mechanisms of tinnitus have been made in the last decade. Although tinnitus may be induced by a peripheral insult, the tinnitus generators are found mainly centrally, in and around the primary auditory cortex as well as in many non-auditory higher-order processing centers. Reduced input to the auditory nerve shifts the balance of central excitation and inhibition, and this may lead to hyperactivity, increased bursting activity and increased synchrony. This view is consistent with the multifactorial nature of tinnitus, which involves auditory, attentional, memory, and emotional systems (Kaltenbach, 2011).

The current view on tinnitus therefore is that it is a symptom encompassing a distributed network across the peripheral and central auditory system. Many studies would indicate that the restoration of cochlear output to the brain should also abolish tinnitus. Preliminary evidence reporting benefit from hearing aids and cochlear implants for tinnitus support this view.

Recent novel findings may open perspectives for new therapeutic approaches on molecular level (e.g., intracochlear application of NMDA antagonists, modulation of microtubule associated proteins molecular pathway, GABA modulation); on a systemic level (behavioral strategies, transcranial magnetic stimulation); “hybrid” solutions that would involve synergistic action of pharmacotherapy and Vagal Nerve Stimulation (Bojic et al., 2017) and lastly the intracochlear pharmacological interventions supported by a non-specific, mostly anxiolytic pharmacotherapy (Guitton, 2012). Factors that determine the phase of tinnitus pathophysiological evolution (initiation or maintenance), the level (molecular or systemic), and the mechanism (neurotransmission or neuromodulation) (Guitton, 2012) will in the future determine the therapeutic approach. The therapy of tinnitus will have to be strictly individualized, with an assessment protocol that would define tinnitus in the sense of the phase (chronicity), level of lesion (peripheral or central) and whenever possible – the mechanism of tinnitus maintenance. This approach in tinnitus evaluation will engage specific multidisciplinary teams whose collaboration will have as a center the subjective wellness and improvement of tinnitus patients.

## Author Contributions

HH conceived and designed this study and had contributions to all its stages. HH and SR performed the database searches and abstract screening. HH, SR, TB, and AS performed manual searches and data extraction from the included records, they contributed equally to all other stages of the manuscript development, drafted and revised the manuscript. DH contributed significantly to the final design and intellectual contents of the manuscript. SR created appendices. SR and AS created list of references in EndNote. JP and DH provided consultative advice and revised the final manuscript. DH, HH, and AS revised the manuscript to address reviewer’s comments and to review the English grammar.

## Conflict of Interest Statement

The authors declare that the research was conducted in the absence of any commercial or financial relationships that could be construed as a potential conflict of interest.

## Footnotes

1 https://clinicaltrials.gov/ct2/show/NCT02315508?term=QUIET-1&rank=1

2 https://autifony.com/wp-content/uploads/2017/10/AUTIFONY-CLARITY-1-RESULTS-08-Aug-2016-FINAL.pdf
