# Supplementary material for: Pathophysiology of Subjective Tinnitus: Triggers and Maintenance
Source: Front Neurosci. 2018 Nov 27;12:866. doi: 10.3389/fnins.2018.00866 (PMC6277522; doi:10.3389/fnins.2018.00866)
Supplement: Supplementary file 1 [file Table_1.DOCX]

**Supplementary Material 1**

**Example search PubMed**

(subjective[All Fields] AND chronic[All Fields] AND ("tinnitus"[MeSH Terms] OR "tinnitus"[All Fields])) AND (("physiopathology"[Subheading] OR "physiopathology"[All Fields] OR "pathophysiology"[All Fields]) OR (patho[All Fields] OR patho'g'enie[All Fields] OR patho1[All Fields] OR patho2[All Fields] OR pathoadapative[All Fields] OR pathoadapt[All Fields] OR pathoadaptation[All Fields] OR pathoadaptative[All Fields] OR pathoadapted[All Fields] OR pathoadaption[All Fields] OR pathoadaptive[All Fields] OR pathoaetiological[All Fields] OR pathoaetiologies[All Fields] OR pathoaetiology[All Fields] OR pathoanathomy[All Fields] OR pathoanatomen[All Fields] OR pathoanatomic[All Fields] OR pathoanatomic'[All Fields] OR pathoanatomical[All Fields] OR pathoanatomically[All Fields] OR pathoanatomie[All Fields] OR pathoanatomies[All Fields] OR pathoanatomisch[All Fields] OR pathoanatomische[All Fields] OR pathoanatomischen[All Fields] OR pathoanatomischer[All Fields] OR pathoanatomisches[All Fields] OR pathoanatomist[All Fields] OR pathoanatomists[All Fields] OR pathoanatomize[All Fields] OR pathoanatomo[All Fields] OR pathoanatomy[All Fields] OR pathoanatootmic[All Fields] OR pathoangiogenesis[All Fields] OR pathoangiogenic[All Fields] OR pathoantigen[All Fields] OR pathoantigenic[All Fields] OR pathoantigenicity[All Fields] OR pathoantigenos[All Fields] OR pathoantigens[All Fields] OR pathoantigens'[All Fields] OR pathoarchitectonic[All Fields] OR pathoarchitectonics[All Fields] OR pathoarray[All Fields] OR pathoarrays[All Fields] OR pathoarrays'[All Fields] OR pathobacteria[All Fields] OR pathobacteriological[All Fields] OR pathobactyper[All Fields] OR pathobasiliensis[All Fields] OR pathobehavioral[All Fields] OR pathobilogy[All Fields] OR pathobio[All Fields] OR pathobioants[All Fields] OR pathobiobiology[All Fields] OR pathobiocchemistry[All Fields] OR pathobiocenose[All Fields] OR pathobiocenoses[All Fields] OR pathobiocenosis[All Fields] OR pathobiochem[All Fields] OR pathobiochemical[All Fields] OR pathobiochemically[All Fields] OR pathobiochemie[All Fields] OR pathobiochemie1[All Fields] OR pathobiochemische[All Fields] OR pathobiochemischen[All Fields] OR pathobiochemischer[All Fields] OR pathobiochemistruy[All Fields] OR pathobiochemistry[All Fields] OR pathobiochemistrycharite[All Fields] OR pathobiochemistrygerman[All Fields] OR pathobiochemistrymolecular[All Fields] OR pathobiochemistryotto[All Fields] OR pathobiochemisty[All Fields] OR pathobiodynamics[All Fields] OR pathobioenergetics[All Fields] OR pathobiogenesis[All Fields] OR pathobiogenetic[All Fields] OR pathobiografical[All Fields] OR pathobiographical[All Fields] OR pathobiographie[All Fields] OR pathobiography[All Fields] OR pathobiographyand[All Fields] OR pathobioilogy[All Fields] OR pathobioinformatics[All Fields] OR pathobiokemiai[All Fields] OR pathobiol[All Fields] OR pathobiolgical[All Fields] OR pathobiolgoy[All Fields] OR pathobiolgy[All Fields] OR pathobioloby[All Fields] OR pathobiologiaja[All Fields] OR pathobiologial[All Fields] OR pathobiologic[All Fields] OR pathobiological[All Fields] OR pathobiologically[All Fields] OR pathobiologie[All Fields] OR pathobiologies[All Fields] OR pathobiologisch[All Fields] OR pathobiologische[All Fields] OR pathobiologist[All Fields] OR pathobiologists[All Fields] OR pathobiologoy[All Fields] OR pathobiolography[All Fields] OR pathobiology[All Fields] OR pathobiology'[All Fields] OR pathobiologygraduate[All Fields] OR pathobiologymedicine[All Fields] OR pathobiologyontario[All Fields] OR pathobiologyschool[All Fields] OR pathobiologyt[All Fields] OR pathobiologytufts[All Fields] OR pathobiologyuniversity[All Fields] OR pathobiologyvetsuisse[All Fields] OR pathobiologyvirginia[All Fields] OR pathobiologyy[All Fields] OR pathobioloigcal[All Fields] OR pathobiololgy[All Fields] OR pathobiolology[All Fields] OR pathobioloy[All Fields] OR pathobiome[All Fields] OR pathobiome'[All Fields] OR pathobiomechanic[All Fields] OR pathobiomechanical[All Fields] OR pathobiomechanics[All Fields] OR pathobiomechanik[All Fields] OR pathobiomes[All Fields] OR pathobiomolecular[All Fields] OR pathobiont[All Fields] OR pathobiontic[All Fields] OR pathobionts[All Fields] OR pathobionts'[All Fields] OR pathobioses[All Fields] OR pathobiosis[All Fields] OR pathobiota[All Fields] OR pathobiotic[All Fields] OR pathobiotype[All Fields] OR pathobiotypes[All Fields] OR pathoblast[All Fields] OR pathoblocker[All Fields] OR pathoblockers[All Fields] OR pathocascade[All Fields] OR pathocascades[All Fields] OR pathocausal[All Fields] OR pathocellular[All Fields] OR pathocenose[All Fields] OR pathocenoses[All Fields] OR pathocenosis[All Fields] OR pathocentric[All Fields] OR pathocentrism[All Fields] OR pathocharacter[All Fields] OR pathocharacteriological[All Fields] OR pathocharacteristic[All Fields] OR pathocharacterologic[All Fields] OR pathocharacterological[All Fields] OR pathocharacterology[All Fields] OR pathocharakterologie[All Fields] OR pathochemical[All Fields] OR pathochemie[All Fields] OR pathochemische[All Fields] OR pathochemistry[All Fields] OR pathochimie[All Fields] OR pathochip[All Fields] OR pathochronia[All Fields] OR pathochronic[All Fields] OR pathochronology[All Fields] OR pathochrony[All Fields] OR pathocidicus[All Fields] OR pathocidin[All Fields] OR pathocidini[All Fields] OR pathocity[All Fields] OR pathoclinical[All Fields] OR pathoclise[All Fields] OR pathoclisis[All Fields] OR pathoclitic[All Fields] OR pathoclosis[All Fields] OR pathocmu[All Fields] OR pathocoenoses[All Fields] OR pathocoenosis[All Fields] OR pathocoenosis'[All Fields] OR pathocom[All Fields] OR pathocon[All Fields] OR pathoconformly[All Fields] OR pathoconnectivity[All Fields] OR pathoconnectomics[All Fields] OR pathoconstellation[All Fields] OR pathocronia[All Fields] OR pathocycles[All Fields] OR pathocyclia[All Fields] OR pathocytologic[All Fields] OR pathocytological[All Fields] OR pathocytologists[All Fields] OR pathod[All Fields] OR pathodb[All Fields] OR pathodemographic[All Fields] OR pathodenese[All Fields] OR pathodenic[All Fields] OR pathodevelopmental[All Fields] OR pathodg[All Fields] OR pathodgenesis[All Fields] OR pathodgentic[All Fields] OR pathodiagnostic[All Fields] OR pathodiagnostica[All Fields] OR pathodiagnostik[All Fields] OR pathodiagnosztikus[All Fields] OR pathodontic[All Fields] OR pathodx[All Fields] OR pathodyn[All Fields] OR pathodynamic[All Fields] OR pathodynamical[All Fields] OR pathodynamics[All Fields] OR pathodynamik[All Fields] OR pathodynamism[All Fields] OR pathoecology[All Fields] OR pathoeener[All Fields] OR pathoeenic[All Fields] OR pathoegenesis[All Fields] OR pathoegnesis[All Fields] OR pathoehn[All Fields] OR pathoelectrophysiologic[All Fields] OR pathoelectrophysiological[All Fields] OR pathoelectrophysiology[All Fields] OR pathoembriology[All Fields] OR pathoembryogenesis[All Fields] OR pathoembryogenic[All Fields] OR pathoembryological[All Fields] OR pathoembryology[All Fields] OR pathoenes[All Fields] OR pathoenie[All Fields] OR pathoens[All Fields] OR pathoenvironment[All Fields] OR pathoepidemiologic[All Fields] OR pathoepidemiological[All Fields] OR pathoepidemiologically[All Fields] OR pathoepidemiology[All Fields] OR pathoepigenetic[All Fields] OR pathoergont[All Fields] OR pathoethiological[All Fields] OR pathoethiology[All Fields] OR pathoetiogenesis[All Fields] OR pathoetiologic[All Fields] OR pathoetiological[All Fields] OR pathoetiologically[All Fields] OR pathoetiologies[All Fields] OR pathoetiology[All Fields] OR pathofens[All Fields] OR pathofibrogenesis[All Fields] OR pathofinder[All Fields] OR pathofindertrade[All Fields] OR pathofisiology[All Fields] OR pathofiziologiai[All Fields] OR pathofobia[All Fields] OR pathoforensic[All Fields] OR pathoforms[All Fields] OR pathofshutcm[All Fields] OR pathofunction[All Fields] OR pathofunctional[All Fields] OR pathofunctions[All Fields] OR pathofunktion[All Fields] OR pathofyrsiologie[All Fields] OR pathofysiogische[All Fields] OR pathofysiol[All Fields] OR pathofysiologi[All Fields] OR pathofysiologic[All Fields] OR pathofysiological[All Fields] OR pathofysiologicka[All Fields] OR pathofysiologicky[All Fields] OR pathofysiologickych[All Fields] OR pathofysiologie[All Fields] OR pathofysiologii[All Fields] OR pathofysiologisch[All Fields] OR pathofysiologische[All Fields] OR pathofysiology[All Fields] OR pathofyziologicke[All Fields] OR pathofyziologii[All Fields] OR pathog[All Fields] OR pathog'en'eit'e[All Fields] OR pathog'en'ese[All Fields] OR pathog'en'etique[All Fields] OR pathog'ene[All Fields] OR pathog'enes[All Fields] OR pathog'enie[All Fields] OR pathog'enique[All Fields] OR pathog'eniques[All Fields] OR pathog'enit'e[All Fields] OR pathog9enese[All Fields] OR pathog9enie[All Fields] OR pathogaenetic[All Fields] OR pathogaenicity[All Fields] OR pathogaenique[All Fields] OR pathogalvanismus[All Fields] OR pathoganesis[All Fields] OR pathoge[All Fields] OR pathoge'ne[All Fields] OR pathoge'nes[All Fields] OR pathogebie[All Fields] OR pathogeen[All Fields] OR pathogeenaanbod[All Fields] OR pathogeenie[All Fields] OR pathogeensis[All Fields] OR pathogeese[All Fields] OR pathogeetic[All Fields] OR pathogeic[All Fields] OR pathogeie[All Fields] OR pathogemnique[All Fields] OR pathogen[All Fields] OR pathogen'[All Fields] OR pathogen'ese[All Fields] OR pathogen'eze[All Fields] OR pathogen's[All Fields] OR pathogen1[All Fields] OR pathogen1,2[All Fields] OR pathogenase[All Fields] OR pathogenasis[All Fields] OR pathogenaspergillus[All Fields] OR pathogenassociated[All Fields] OR pathogenatic[All Fields] OR pathogenb[All Fields] OR pathogenbecause[All Fields] OR pathogenbiology[All Fields] OR pathogenc[All Fields] OR pathogencandida[All Fields] OR pathogencaused[All Fields] OR pathogenccity[All Fields] OR pathogenci[All Fields] OR pathogencitiy[All Fields] OR pathogencity[All Fields] OR pathogencombat[All Fields] OR pathogenderived[All Fields] OR pathogendetection[All Fields] OR pathogendiagnostics[All Fields] OR pathogendiagnostik[All Fields] OR pathogene[All Fields] OR pathogene'[All Fields] OR pathogene's[All Fields] OR pathogene'sis[All Fields] OR pathogenecg[All Fields] OR pathogenecities[All Fields] OR pathogenecity[All Fields] OR pathogenectic[All Fields] OR pathogenecy[All Fields] OR pathogenedis[All Fields] OR pathogenei[All Fields] OR pathogeneia[All Fields] OR pathogeneian[All Fields] OR pathogeneias[All Fields] OR pathogeneic[All Fields] OR pathogeneicite[All Fields] OR pathogeneicities[All Fields] OR pathogeneicity[All Fields] OR pathogeneiity[All Fields] OR pathogeneis[All Fields] OR pathogeneisis[All Fields] OR pathogeneiss[All Fields] OR pathogeneite[All Fields] OR pathogeneity[All Fields] OR pathogenem[All Fields] OR pathogenen[All Fields] OR pathogenene[All Fields] OR pathogenenesis[All Fields] OR pathogenenic[All Fields] OR pathogenenicity[All Fields] OR pathogenenie[All Fields] OR pathogenenique[All Fields] OR pathogenenis[All Fields] OR pathogenens[All Fields] OR pathogenensis[All Fields] OR pathogenentic[All Fields] OR pathogeneous[All Fields] OR pathogener[All Fields] OR pathogeneric[All Fields] OR pathogenes[All Fields] OR pathogenes'[All Fields] OR pathogenesa[All Fields] OR pathogenesc[All Fields] OR pathogenese[All Fields] OR pathogenesefaktor[All Fields] OR pathogeneseforschung[All Fields] OR pathogeneseis[All Fields] OR pathogenesekonzepte[All Fields] OR pathogenesemechanismen[All Fields] OR pathogenesemodelle[All Fields] OR pathogenesen[All Fields] OR pathogeneseorientierte[All Fields] OR pathogeneseorientierten[All Fields] OR pathogeneseos[All Fields] OR pathogeneses[All Fields] OR pathogenesesis[All Fields] OR pathogenesewege[All Fields] OR pathogenesi[All Fields] OR pathogenesic[All Fields] OR pathogenesics[All Fields] OR pathogenesiis[All Fields] OR pathogenesin[All Fields] OR pathogenesis[All Fields] OR pathogenesis'[All Fields] OR pathogenesis'eben[All Fields] OR pathogenesis'enek[All Fields] OR pathogenesis'eroel[All Fields] OR pathogenesis,[All Fields] OR pathogenesisand[All Fields] OR pathogenesisaof[All Fields] OR pathogenesisassociated[All Fields] OR pathogenesisbased[All Fields] OR pathogenesise[All Fields] OR pathogenesiseben[All Fields] OR pathogenesisehez[All Fields] OR pathogenesisenek[All Fields] OR pathogenesisere[All Fields] OR pathogenesiserol[All Fields] OR pathogenesises[All Fields] OR pathogenesisevel[All Fields] OR pathogenesisi[All Fields] OR pathogenesisis[All Fields] OR pathogenesisity[All Fields] OR pathogenesisly[All Fields] OR pathogenesisof[All Fields] OR pathogenesisre[All Fields] OR pathogenesisrelated[All Fields] OR pathogenesiss[All Fields] OR pathogenesissquareof[All Fields] OR pathogenesity[All Fields] OR pathogeness[All Fields] OR pathogenestic[All Fields] OR pathogenests[All Fields] OR pathogenesu[All Fields] OR pathogeneswis[All Fields] OR pathogenesy[All Fields] OR pathogenetc[All Fields] OR pathogeneteic[All Fields] OR pathogenethic[All Fields] OR pathogenetic[All Fields] OR pathogenetic's[All Fields] OR pathogenetical[All Fields] OR pathogeneticall[All Fields] OR pathogenetically[All Fields] OR pathogeneticaly[All Fields] OR pathogenetice[All Fields] OR pathogenetiche[All Fields] OR pathogenetiches[All Fields] OR pathogeneticheskaia[All Fields] OR pathogeneticheskie[All Fields] OR pathogeneticheskogo[All Fields] OR pathogeneticity[All Fields] OR pathogeneticka[All Fields] OR pathogeneticke[All Fields] OR pathogeneticky[All Fields] OR pathogeneticlink[All Fields] OR pathogeneticly[All Fields] OR pathogenetico[All Fields] OR pathogenetics[All Fields] OR pathogenetics'[All Fields] OR pathogeneticsymptoms[All Fields] OR pathogenetik[All Fields] OR pathogenetikai[All Fields] OR pathogenetikally[All Fields] OR pathogenetique[All Fields] OR pathogenetiques[All Fields] OR pathogenetis[All Fields] OR pathogenetisch[All Fields] OR pathogenetische[All Fields] OR pathogenetischem[All Fields] OR pathogenetischen[All Fields] OR pathogenetischer[All Fields] OR pathogenetisches[All Fields] OR pathogenetisk[All Fields] OR pathogenetiske[All Fields] OR pathogenetitto[All Fields] OR pathogenetix[All Fields] OR pathogenetsis[All Fields] OR pathogenety[All Fields] OR pathogenetycznych[All Fields] OR pathogeneus[All Fields] OR pathogenez[All Fields] OR pathogeneza[All Fields] OR pathogeneze[All Fields] OR pathogenezis'ehez[All Fields] OR pathogenezise[All Fields] OR pathogenezisehez[All Fields] OR pathogenezisenek[All Fields] OR pathogeneziserol[All Fields] OR pathogenezu[All Fields] OR pathogenfinder[All Fields] OR pathogenfor[All Fields] OR pathogenfree[All Fields] OR pathogenfreie[All Fields] OR pathogenfreien[All Fields] OR pathogenfreier[All Fields] OR pathogengesis[All Fields] OR pathogenhelminthosporium[All Fields] OR pathogenhost[All Fields] OR pathogeni[All Fields] OR pathogenia[All Fields] OR pathogeniat[All Fields] OR pathogenic[All Fields] OR pathogenic'[All Fields] OR pathogenic'mycobacterium[All Fields] OR pathogenica[All Fields] OR pathogenicagents[All Fields] OR pathogenical[All Fields] OR pathogenically[All Fields] OR pathogenicaly[All Fields] OR pathogeniccd4[All Fields] OR pathogeniccontribution[All Fields] OR pathogenice[All Fields] OR pathogenicescherichia[All Fields] OR pathogenicety[All Fields] OR pathogenicforms[All Fields] OR pathogenicicity[All Fields] OR pathogenicicy[All Fields] OR pathogenicin[All Fields] OR pathogeniciry[All Fields] OR pathogenicit[All Fields] OR pathogenicit'e[All Fields] OR pathogenicita[All Fields] OR pathogenicite[All Fields] OR pathogeniciteit[All Fields] OR pathogenicities[All Fields] OR pathogenicitiy[All Fields] OR pathogenicity[All Fields] OR pathogenicity'[All Fields] OR pathogenicityof[All Fields] OR pathogenicityon[All Fields] OR pathogenicityseems[All Fields] OR pathogeniciy[All Fields] OR pathogenics[All Fields] OR pathogenicseoul[All Fields] OR pathogenicsporothrixspp[All Fields] OR pathogenicty[All Fields] OR pathogenicvibrio[All Fields] OR pathogenicyars2[All Fields] OR pathogenid[All Fields] OR pathogenie[All Fields] OR pathogenies[All Fields] OR pathogeniese[All Fields] OR pathogeninactivation[All Fields] OR pathogeninaktivierte[All Fields] OR pathogeninduced[All Fields] OR pathogeninfected[All Fields] OR pathogenio[All Fields] OR pathogeniobiology[All Fields] OR pathogenioc[All Fields] OR pathogeniology[All Fields] OR pathogenique[All Fields] OR pathogeniques[All Fields] OR pathogenis[All Fields] OR pathogenische[All Fields] OR pathogenischen[All Fields] OR pathogenisis[All Fields] OR pathogenisity[All Fields] OR pathogenism[All Fields] OR pathogeniss[All Fields] OR pathogenita[All Fields] OR pathogenitaet[All Fields] OR pathogenital[All Fields] OR pathogenitasa[All Fields] OR pathogenitasanak[All Fields] OR pathogenitat[All Fields] OR pathogenitats[All Fields] OR pathogenitatsbestimmung[All Fields] OR pathogenitatserhaltung[All Fields] OR pathogenitatsfaktor[All Fields] OR pathogenitatsfaktoren[All Fields] OR pathogenitatsfaktors[All Fields] OR pathogenitatsfrage[All Fields] OR pathogenitatsinseln[All Fields] OR pathogenitatskriterien[All Fields] OR pathogenitatsmechanismen[All Fields] OR pathogenitatsmerkmale[All Fields] OR pathogenitatsnachweis[All Fields] OR pathogenitatsproblem[All Fields] OR pathogenitatsprufungen[All Fields] OR pathogenitatsteste[All Fields] OR pathogenite[All Fields] OR pathogeniteit[All Fields] OR pathogenitic[All Fields] OR pathogenitically[All Fields] OR pathogeniticy[All Fields] OR pathogenities[All Fields] OR pathogenity[All Fields] OR pathogenius[All Fields] OR pathogenization[All Fields] OR pathogenize[All Fields] OR pathogenized[All Fields] OR pathogenizing[All Fields] OR pathogenizing'[All Fields] OR pathogenlss[All Fields] OR pathogenm[All Fields] OR pathogenmip[All Fields] OR pathogenmiper[All Fields] OR pathogenna[All Fields] OR pathogennach[All Fields] OR pathogenni[All Fields] OR pathogennich[All Fields] OR pathogenniho[All Fields] OR pathogennosema[All Fields] OR pathogennye[All Fields] OR pathogennykh[All Fields] OR pathogeno[All Fields] OR pathogenobiology[All Fields]))
